# Obesity as a mortality risk factor in the medical ward: a case control study

**DOI:** 10.1186/s12902-021-00912-5

**Published:** 2022-01-06

**Authors:** Shelly Soffer, Eyal Zimlichman, Benjamin S. Glicksberg, Orly Efros, Matthew A. Levin, Robert Freeman, David L. Reich, Eyal Klang

**Affiliations:** 1 Internal Medicine B, Assuta Medical Center, Ashdod, Israel; 2 Ben-Gurion University of the Negev, Be’er Sheva, Israel; 3 Hospital Management, Sheba Medical Center, Tel Hashomer, Israel; 4 Sackler Medical School, Tel Aviv University, Tel Aviv, Israel; 5 Sheba Talpiot Medical Leadership Program, Tel Hashomer, Israel; 6 Hasso Plattner Institute for Digital Health at Mount Sinai, Icahn School of Medicine at Mount Sinai, New York, NY USA; 7 Department of Genetics and Genomic Sciences, Icahn School of Medicine at Mount Sinai, New York, NY USA; 8 Thrombosis & Hemostasis Unit, Sheba Medical Center, Tel Hashomer, Israel; 9 Institute for Healthcare Delivery Science, Department of Population Health Science and Policy, Icahn School of Medicine at Mount Sinai, New York, USA; 10 Department of Anesthesiology, Perioperative and Pain Medicine, Icahn School of Medicine at Mount Sinai, New York, USA; 11 Department of Diagnostic Imaging, Sheba Medical Center, Tel Hashomer, Israel

**Keywords:** Obesity, Hospital mortality, COVID-19, SARS-CoV-2

## Abstract

**Background:**

Research regarding the association between severe obesity and in-hospital mortality is inconsistent. We evaluated the impact of body mass index (BMI) levels on mortality in the medical wards. The analysis was performed separately before and during the COVID-19 pandemic.

**Methods:**

We retrospectively retrieved data of adult patients admitted to the medical wards at the Mount Sinai Health System in New York City. The study was conducted between January 1, 2011, to March 23, 2021. Patients were divided into two sub-cohorts: pre-COVID-19 and during-COVID-19. Patients were then clustered into groups based on BMI ranges. A multivariate logistic regression analysis compared the mortality rate among the BMI groups, before and during the pandemic.

**Results:**

Overall, 179,288 patients were admitted to the medical wards and had a recorded BMI measurement. 149,098 were admitted before the COVID-19 pandemic and 30,190 during the pandemic. Pre-pandemic, multivariate analysis showed a “J curve” between BMI and mortality. Severe obesity (BMI > 40) had an aOR of 0.8 (95% CI:0.7–1.0, *p* = 0.018) compared to the normal BMI group. In contrast, during the pandemic, the analysis showed a “U curve” between BMI and mortality. Severe obesity had an aOR of 1.7 (95% CI:1.3–2.4, *p* < 0.001) compared to the normal BMI group.

**Conclusions:**

Medical ward patients with severe obesity have a lower risk for mortality compared to patients with normal BMI. However, this does not apply during COVID-19, where obesity was a leading risk factor for mortality in the medical wards. It is important for the internal medicine physician to understand the intricacies of the association between obesity and medical ward mortality.

**Supplementary Information:**

The online version contains supplementary material available at 10.1186/s12902-021-00912-5.

## Background

Obesity has reached epidemic proportions worldwide. The prevalence of obesity is up to 40% among adults in the United States [1]. It is well recognized that individuals with obesity are more likely to suffer from diabetes, cardiovascular disease, and certain malignancies [2]. Individuals with obesity require more hospital admissions compared with the non-obese population [3].

There is a strong association between body weight and mortality in the general population [4]. However, controversy exists regarding the effect of severe obesity on in-hospital mortality. Some studies have shown that obesity reduces overall inpatient mortality risk, a phenomenon termed as the “obesity paradox” [5–7], while others demonstrated an increased inpatient mortality risk [8, 9]. However, these studies included a small number of patients with severe obesity and focused on specific illnesses.

On March 11th, the World Health Organization declared the COVID-19 outbreak a pandemic. Shortly after, several risk factors for disease severity and mortality were determined [10, 11]. Severe obesity was shown to be a significant independent risk factor for in-hospital mortality from COVID-19 [12–14].

In this study, we evaluated the impact of BMI levels on mortality in the medical wards. The analysis was performed separately before and during the COVID-19 pandemic.

## Methods

### Study setting and data source

An Institutional Review Board (IRB) approval was granted for this retrospective cohort study. The IRB committee waived informed consent.

We identified consecutive admissions to five hospitals in the Mount Sinai Health System, NY, USA (Mount Sinai Hospital, Mount Sinai Brooklyn, Mount Sinai Queens, Mount Sinai Morningside, and Mount Sinai West). Electronic health record (EHR) data were extracted from EPIC (Epic Systems Corporation, Verona WI). The study time frame was from January 1, 2011, to March 23, 2021.

### Study design

#### Population

We included patients admitted to the medical wards with a measurement of Body Mass Index (BMI). We excluded patients younger than 18 years old. We also excluded patients admitted to the intensive care unit (ICU).

The date March 1, 2020 was used to differentiate between two sub-cohorts: pre-COVID-19 pandemic and during-COVID-19 pandemic. This date was selected according to the time when COVID-19 pandemic started to rise, and the number of hospitalizations rapidly increased [15]. Patients that were hospitalized before March 1st but remained in the hospital after this date were included in the pre-COVID cohort.

#### Variables

The primary outcome was the association between BMI levels and all-cause in-hospital mortality. Covariates of interest included: age, sex, race, and comorbidities. Comorbidities were coded using the International Classification of Diseases, 10th Revision, Clinical Modification (ICD-10-CM), and grouped using the Healthcare Cost and Utilization Project diagnostic clinical classification software (CCS) [17]. They included: coronary artery disease (CAD), congestive heart failure (CHF), diabetes mellitus (DM), hypertension (HTN), chronic kidney disease (CKD), chronic obstructive pulmonary disease (COPD), and cancer. ICD-10 coded primary diagnoses at discharge were extracted from the EHR. Data regarding race was incomplete and in 31% of the patients this variable was categorized as “other”. Data relating ethnicity was mostly missing and thus was not included in the analysis.

Data regarding SARS-CoV-2 nasopharyngeal swab polymerase chain reaction (PCR) tests were retrieved.

### BMI groups selection

Recorded BMI measurements were retrieved from the EHR. Patients were clustered into groups based on BMI measurements: < 18.5 kg/m^2^, 18.5–24.9 kg/m^2^, 25–29.9 kg/m^2^, 30–34.9 kg/m^2^, 35–39.9 kg/m^2^, ≥ 40 kg/m^2^ [16]. Severe obesity was defined as a BMI above 40 kg/m^2^ [16].

### Statistical analysis

Descriptive statistics were reported for all patient characteristics using means and standard deviation or medians with inter quartile range (IQR) for continuous variables and counts with percentages for categorical variables. Continuous variables were compared using either the unpaired t-test for two variables or 1-way analysis of variance (ANOVA) for more than two variables. Categorical variables were compared using the χ2 test. Separate analyses were performed for the pre-COVID-19 pandemic and the during-COVID-19 pandemic cohorts.

Age distribution curves were plotted for different BMI groups. Gaussian kernel density estimates were fitted to the plots.

We employed the mutual information (MI) formula to identify primary diagnoses associated with severe obesity. Mutual information measures the statistical dependence between the severe obesity group (G) and a given diagnosis (d), as follows:

$$ Mutual\ Information=\sum \sum P\left(G,d\right)\ast Log\ \frac{P\left(G,d\right)}{P(G)P(d)} $$

$$ P\left(G,d\right)= Joint\ probability\ of\ G\  and\ d;P(G)= probability\ of\ G;P(d)= probability\ of\ d. $$

In addition to MI, we also determined odds ratios (OR).

Multivariate logistic regression models compared rates of mortality between the different BMI groups. Separate models were built for the pre-COVID-19 pandemic and during-COVID-19 pandemic cohorts. The models were adjusted for demographics (age, sex, race) and comorbidities (CAD, CHF, DM, HTN, CKD, COPD, cancer). Patients with normal BMI measurements (18.5–24.9 kg/m^2^) were used as reference. Correlation matrices were constructed to assess the possible collinearity between covariates. All covariate correlations were below 0.46 (Appendix Fig. 1A, B). Adjusted odds ratios (aOR) with 95% confidence intervals (CI) were reported.

C-statistic was calculated for the multivariate models. Bootstrapping validations (1000 bootstrap resamples) were used to calculate 95% confidence intervals (CI) for all metrics.

All analyses were conducted with Python (Python software foundation, Version 3.6.5).

## Results

**Fig. 1:**
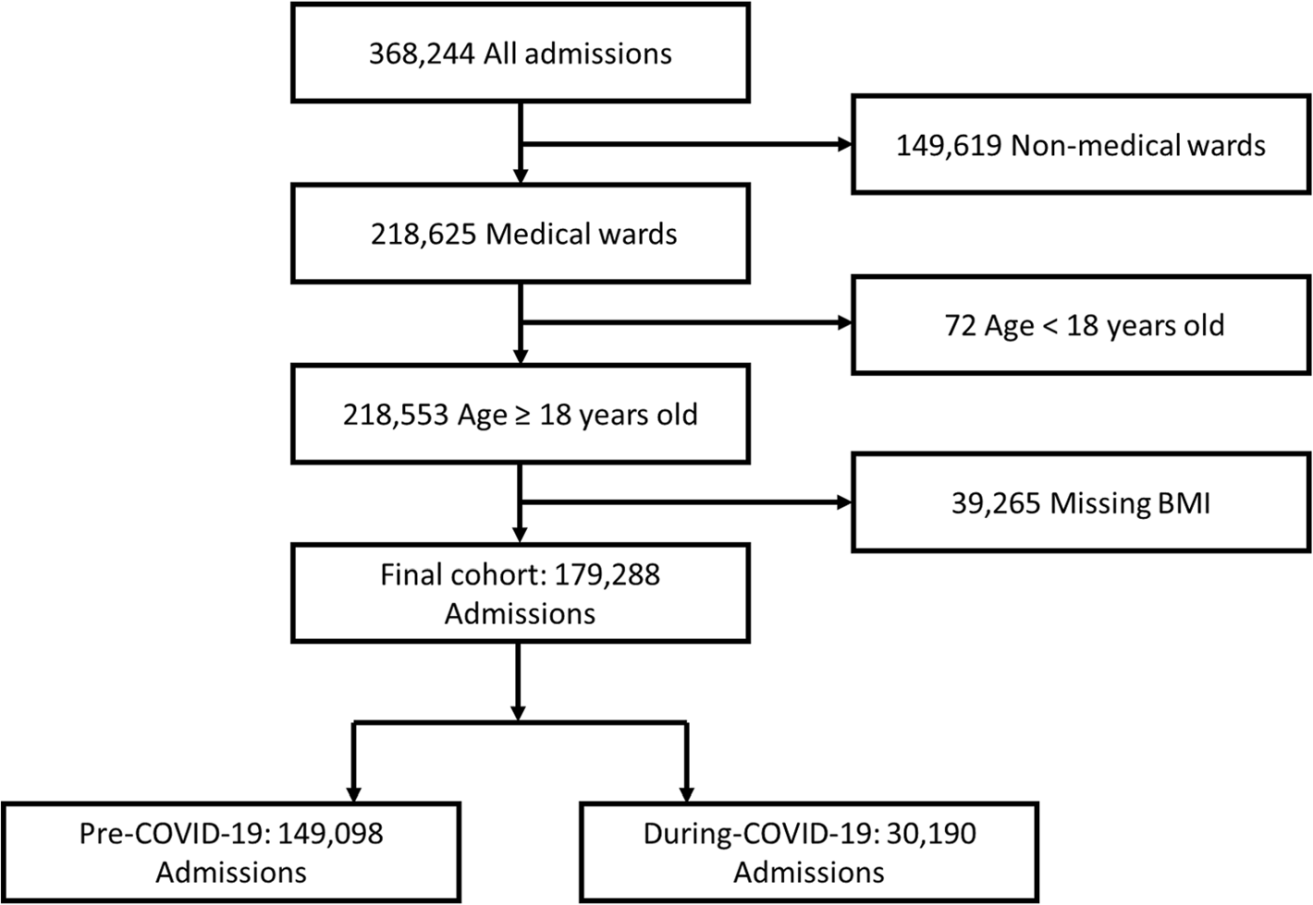
Study flow chart

The cohort included 179,288 patients that were admitted to the medical wards. Of those, 149,098 were hospitalized before the COVID-19 pandemic and 30,190 during the pandemic. Figure 1 presents the study inclusion flow chart.

The median age of the entire cohort was 65 years (IQR 51–78), and 91,312 (50.9%) patients were females (Table 1). The overall in-hospital mortality rate was 2.3% (*n* = 4200). The mortality rate was significantly higher during the COVID-19 pandemic (3.3% versus 2.2%, *P* < 0.001).

**Table 1:** Clinical and demographic characteristics of patient cohort before and during the COVID-19 pandemic (*n* = 179,288)

	Entire Cohort (*n* = 179,288)	pre-COVID-19 (*n* = 149,098, 83.2%)	during-COVID-19 (*n* = 30,190, 16.8%)	*P* value
BMI groups				
< 18.5 kg/m^2^, N. (%)	10,775 (6.0)	8950 (6.0)	1825 (6.0)	< 0.001
18.5–24.9 kg/m^2^, N. (%)	64,757 (36.1)	54,225 (36.4)	10,532 (34.9)	< 0.001
25–29.9 kg/m^2^, N. (%)	52,436 (29.2)	43,426 (29.1)	9010 (29.8)	< 0.001
30–34.9 kg/m^2^, N. (%)	27,576 (15.4)	22,813 (15.3)	4763 (15.8)	< 0.001
35–39.9 kg/m^2^, N. (%)	12,742 (7.1)	10,500 (7.0)	2242 (7.4)	< 0.001
≥ 40 kg/m^2^, N. (%)	11,002 (6.1)	9184 (6.2)	1818 (6.0)	< 0.001
Demographics				
Age, median (IQR), y	65.0 (51.0–78.0)	65.0 (51.0–78.0)	66.0 (53.0–78.0)	< 0.001
Female, N. (%)	91,308 (50.9)	76,785 (51.5)	14,523 (48.1)	< 0.001
Black, N. (%)	48,515 (27.1)	39,362 (26.4)	9153 (30.3)	< 0.001
White, N. (%)	57,700 (32.2)	48,241 (32.4)	9459 (31.3)	< 0.001
Comorbidities				
CAD, N. (%)	44,505 (24.8)	35,740 (24.0)	8765 (29.0)	< 0.001
CHF, N. (%)	40,546 (22.6)	32,995 (22.1)	7551 (25.0)	< 0.001
DM, N. (%)	66,022 (36.8)	53,796 (36.1)	12,226 (40.5)	< 0.001
HTN, N. (%)	92,294 (51.5)	74,896 (50.2)	17,398 (57.6)	< 0.001
CKD, N. (%)	33,031 (18.4)	27,150 (18.2)	5881 (19.5)	< 0.001
COPD, N. (%)	24,788 (13.8)	20,613 (13.8)	4175 (13.8)	0.99
Cancer, N. (%)	51,659 (28.8)	42,591 (28.6)	9068 (30.0)	< 0.001
Past or present smoking, N. (%)	75,186 (41.9)	63,967 (42.9)	11,219 (37.2)	< 0.001
Mortality				
Mortality rate, N. (%)	4200 (2.3)	3210 (2.2)	990 (3.3)	< 0.001

Abbreviations: BMI - body mass index; IQR - interquartile range; CAD – Coronary artery disease; CHF – Congestive heart failure; DM – Diabetes mellitus; HTN – hypertension; CKD – Chronic kidney disease; COPD – Chronic obstructive pulmonary disease

Of the during-COVID-19 cohort, 4602 / 30,190 (15.2%) had a positive in-hospital COVID-19 PCR test. The mortality rate was significantly higher among the PCR positive group: there were 527 patients who died out of 4602 with a positive PCR test (11.5%) in comparison to 463 patients who died out of 25,588 with a non-positive PCR test (1.8%), *P* < 0.001.

### BMI levels

The BMI distribution was right-skewed (Appendix Fig. 2), with a median of 26.1 kg/m^2^ (IQR 22.4–26.1). Obesity (BMI ^3^ 30 kg/m^2^) was found in 28.5% in the pre-COVID-19 and 29.2% in the COVID-19 cohort. Severe obesity (BMI ^3^ 40 kg/m^2^) was found in 6.2% of the pre-COVID-19 and 6.0% in the COVID-19 pandemic cohort.

**Fig. 2:**
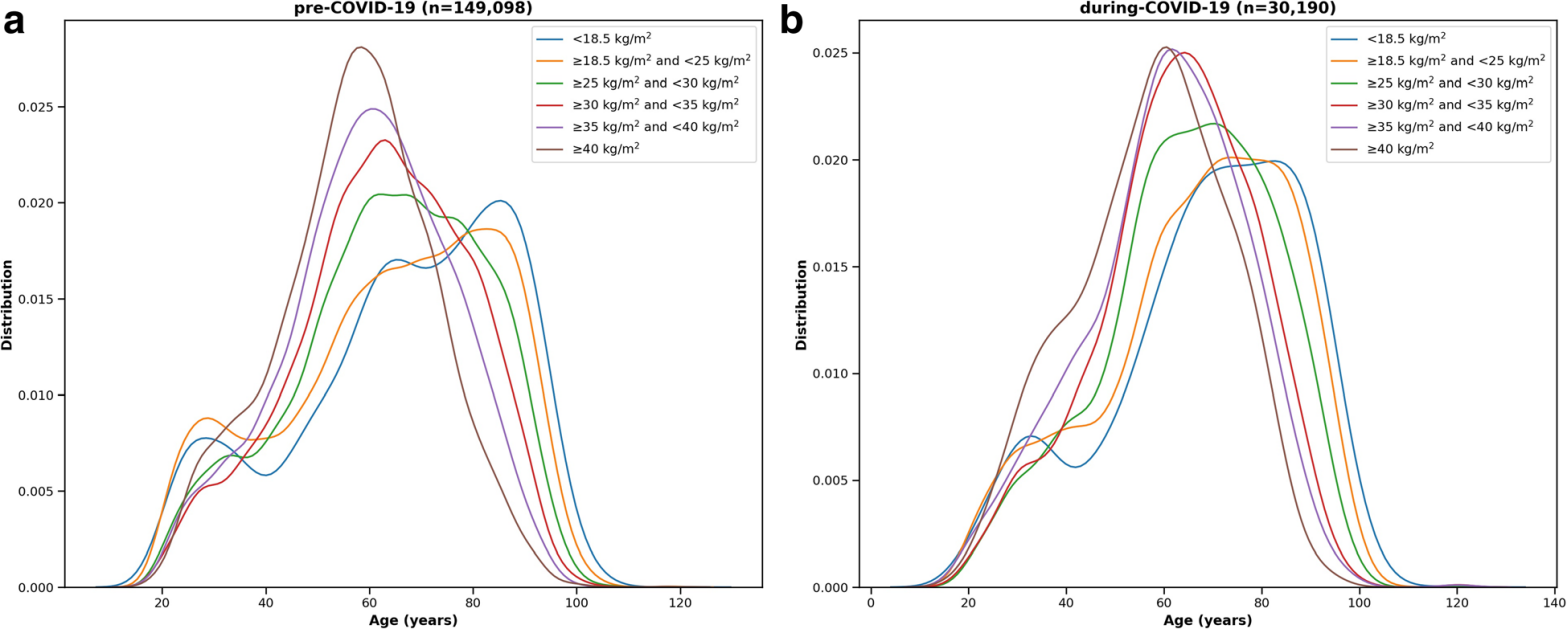
Histograms demonstrating age distributions according to different BMI groups for the **A** pre-COVID-19 cohort and **B** during-COVID-19 cohort

In both cohorts, patients’ median age decreased with higher BMI levels (Fig. 2A, B). Patients with severe obesity had the lowest median age (58 years in the pre-COVID-19 and 59 years in the COVID-19 pandemic cohort) (Appendix Tables 1 and 2). Underweight patients had the highest median age (70 years in the pre-COVID-19 and 71 years in the COVID-19 cohort). Patients with severe obesity were more likely to have comorbidities such as DM, HTN, COPD, and CHF and were less likely to have malignancy.

Appendix Table 3A, B demonstrate the ten most common primary diagnoses in the severe obesity groups. In the pre-COVID-19 cohort, cellulitis had the greatest association with severe obesity (7.5% vs. 2.9%, OR of 2.8, *P* < 0.001), followed by shortness of breath (7.2% vs. 3.3%, OR of 2.3, P < 0.001). Among the during-COVID-19 cohort, shortness of breath had the greatest association with severe obesity (7.4% vs. 3.1%, OR of 2.5, P < 0.001), followed by cellulitis (5.8% vs. 2.2%, OR of 2.8, *p* < 0.001).

### Multivariable models

**Fig. 3:**
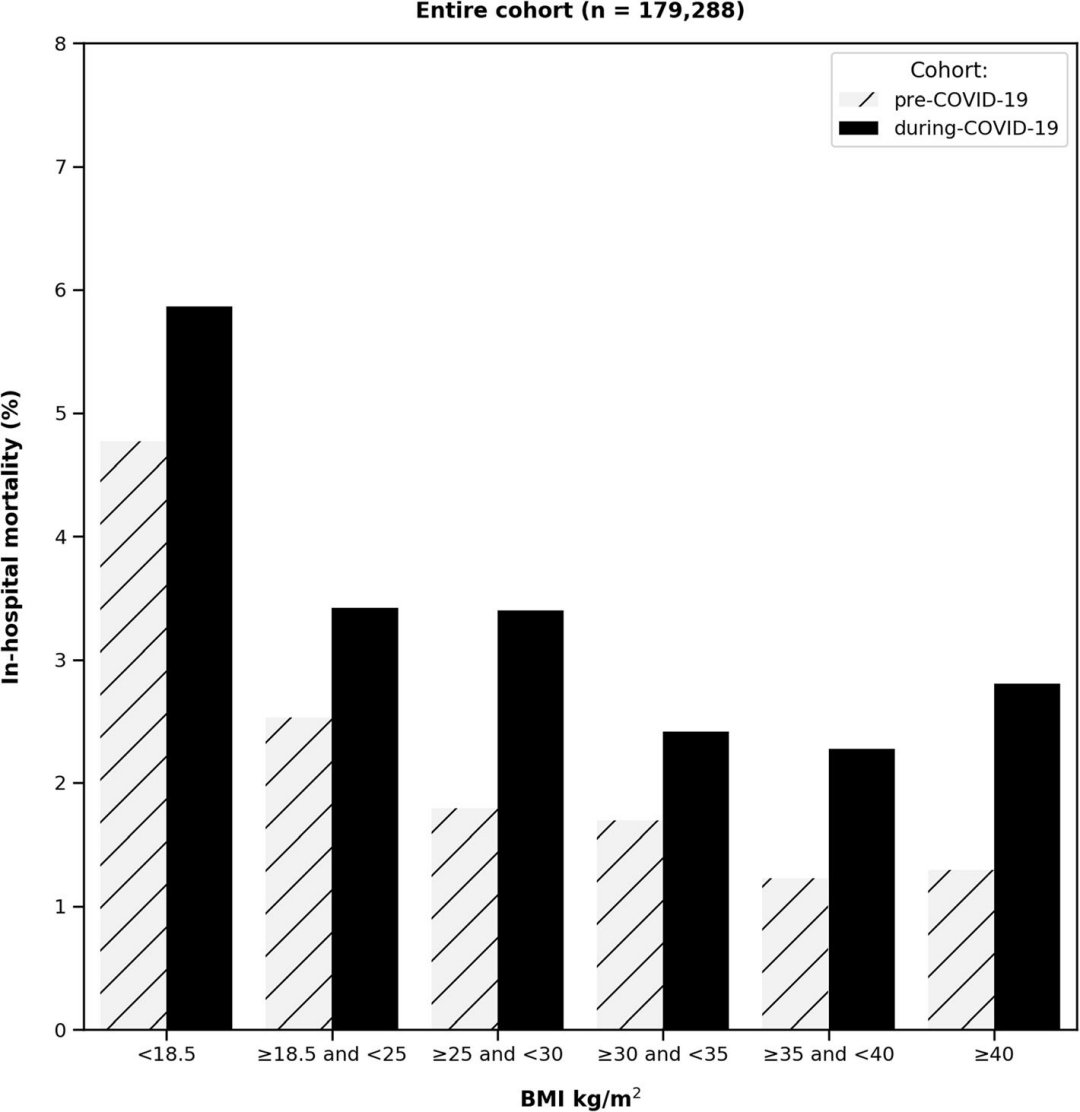
In-hospital mortality rates according to BMI for the pre-COVID-19 cohort and the during-COVID-19 cohort

Crude mortality rates, stratified by BMI levels, are presented in Fig. 3. Mortality rates were higher during the COVID-19 pandemic. Subgroup analysis by age and sex is presented in Appendix Fig. 3A-D.

The multivariable models had C-statistic of 0.71 (95% CI: 0.71–0.72) in the pre-COVID-19 pandemic cohort, and C-statistic of 0.75 (95% CI: 0.73–0.76) in the during-COVID-19 pandemic cohort.

**Fig. 4:**
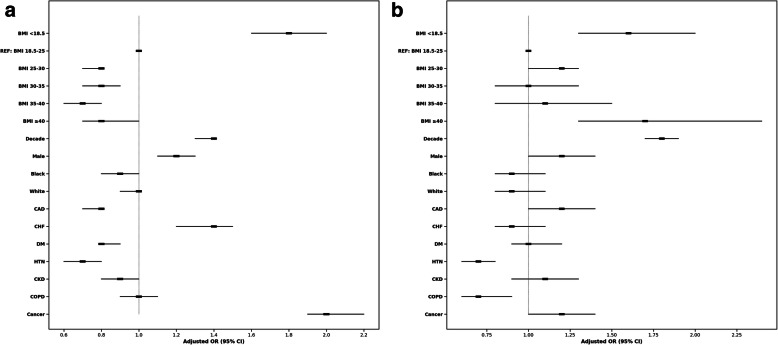
Forest plots presenting multivariable analyses comparing rates of in-hospital mortality between BMI groups for the **A** pre-COVID-19 cohort and **B** during-COVID-19 cohort. The models were adjusted for age decile, male sex, race, CAD, CHF, HTN, DM, CKD, COPD, cancer. Patients with normal BMI measurements (18.5–24.9 kg/m^2^) were used as the reference group. Abbreviations: Coronary artery disease (CAD); Congestive heart failure (CHF); Hypertension (HTN); Diabetes mellitus (DM); Body mass index (BMI); Chronic kidney disease (CKD)

In the pre-COVID-19 cohort, multivariable analysis showed a mild “J curve” (Fig. 4A). Severe obesity had an aOR of 0.8 (95% CI 0.7–1.0, *p* = 0.018) compared to normal BMI. Underweight patients had a significantly higher mortality rate in this cohort compared to the other BMI groups, with an aOR of 1.8 (95% CI: 1.6–2.0, *p* < 0.001).

In contrast, in the during-COVID-19 cohort, multivariable analysis showed a “U curve” (Fig. 4B). Severe obesity had an aOR of 1.7 (95% CI 1.3–2.4, p < 0.001) compared to normal BMI. Underweight patients had the second-highest mortality rate compared to the other BMI groups, with an aOR of 1.6 (95% CI 1.3–2.0, p < 0.001).

## Discussion

This multi-site study evaluated the intricate relationship between BMI levels and mortality in the medical ward.

While obesity is an extremely common morbidity that affects patients in the medical wards, the literature regarding its effect on in-hospital mortality is inconsistent. Previous studies showed obesity to be either associated with a decline, an unchanged, or an elevated risk of mortality. Nevertheless, most studies did not refer to the severe obesity group (BMI ≥ 40 kg/m^2^) [7, 17–21]. Studies that explicitly included severe obesity were based on a limited number of subjects [6, 22–24]. Additionally, they were limited to specific diagnoses such as acute myocardial infarction and intracerebral hemorrhage. In this study, we focused on the all-cause mortality in the medical wards. We included a large multi-site cohort of more than 10,000 patients with severe obesity. We found that before COVID-19, the risk for mortality was higher for underweight patients, lowest for the obese group, and marginally lower for the severely obese. In contrast, in the past year, since the COVID-19 emerged, patients with severe obesity had twice the adjusted risk for mortality. Indeed, during the pandemic, severe obesity was the highest mortality risk factor in the medical wards.

Several mechanisms were proposed to elucidate the “obesity paradox”. It has been theorized that obesity is a marker of nutritional reserve that can be utilized in conditions with high metabolic demands. In contrast, end-stage diseases that inevitably have a high mortality rate and are frequently treated in the medical wards are characterized by cachexia and protein catabolism [25, 26]. In our study, although underweight patients had a lower risk for cardiovascular comorbidities, they were older and had a higher malignancy rate. Elderly individuals are more prone to unintentional weight loss and are at a higher risk for malnutrition [18].

During the COVID-19 pandemic, these factors may not play a similar role. Several mechanisms have been suggested to explain the unique link between obesity and COVID-19 severity [27–31]. The expanded mass of adipose tissue contributes to a prothrombotic state and chronic inflammation [32]. Abnormal cytokine production and microthrombi formation were proposed as key mediators of COVID-19 severity in patients with obesity. Obesity is characterized by physiological lung alteration that can result in ventilatory dysfunction [33]. The decreased functional residual capacity and hypoxemia observed in patients with obesity make them vulnerable to a more severe COVID-19 illness [34]. Additionally, the management of obese patients is further complicated by difficulties in several medical interventions such as intubation and prone positioning [35].

Internal medicine physicians play an important role in the treatment of patients with severe obesity. This clearly applies to the period before as well as during the COVID-19 pandemic. Physicians are educated according to the assumption that obesity is a risk factor for mortality. However, it is important to have a more in-depth understanding of the context of this premise.

Our study has several limitations. This is a retrospective study and may suffer from inherent biases. The two cohorts demonstrated substantial differences in age, comorbidities, and demographics. Although a multivariate regression analysis was performed with adjustment to age and comorbidities, we could not incorporate all possible influencing factors. Therefore, a selective survival bias may remain. Moreover, data on disease severity at presentation was not available in this study. It is possible that physicians tended to hospitalize patients with obesity in a milder condition, influencing the cohort observed prognosis. Although we demonstrated an association between BMI and mortality, a cause-and-effect relationship cannot be deduced between these two variables. Additionally, even though this study was based on a multi-site cohort, it was limited to the New York population. Lastly, we used BMI to define severe obesity. Although indices that reflect adipose tissue distribution were also shown to correlate with COVID-19 severity, they were not available for this work.

## Conclusion

Medical ward patients with severe obesity have a lower risk for mortality compared to patients with normal BMI. However, this does not apply during COVID-19, where obesity was a leading risk factor for mortality in the medical wards. It is important for the internal medicine physician to understand the intricacies of the association between obesity and medical ward mortality.

## Supplementary Information

**Additional file 1.**

## Data Availability

The data used to support the findings of this study are available from the corresponding author upon reasonable request.

## Abbreviations

BMI: Body mass index
IRB: Institutional Review Board
EHR: Electronic health record
ICU: Intensive care unit
CCS: Clinical classification software
CAD: Coronary artery disease
CHF: Congestive heart failure
DM: Diabetes mellitus
HTN: Hypertension
CKD: Chronic kidney disease
COPD: Chronic obstructive pulmonary disease
PCR: Polymerase chain reaction
MI: Mutual information
OR: Odds ratios
CI: Confidence intervals
