# Supplementary material for: Obesity as a mortality risk factor in the medical ward: a case control study
Source: BMC Endocr Disord. 2022 Jan 6;22:13. doi: 10.1186/s12902-021-00912-5 (PMC8733434; doi:10.1186/s12902-021-00912-5)
Supplement: Supplementary file 1 — Additional file 1. [file 12902_2021_912_MOESM1_ESM.docx]

**Supplementary Table 1**: Clinical and demographic characteristics of patients in the pre-COVID-19 cohort, stratified by BMI groups.

|  | **<18.5 kg/m^2^ (n = 8950, 6.0%)** | **18.5 - 25 kg/m^2^ (n = 54,225, 36.4%)** | **25 - 30 kg/m^2^ (n = 43,426, 29.1%)** | **30 - 35 kg/m^2^ (n = 22,813, 15.3%)** | **35 - 40 kg/m^2^ (n = 10,500, 7.0%)** | **≥40 kg/m^2^ (n = 9184, 6.2%)** | **P value** |
| --- | --- | --- | --- | --- | --- | --- | --- |
| **Demographics** | | | | | | | |
| **Age, median (IQR), y** | 70.0 (53.0 - 84.0) | 67.0 (50.0 - 81.0) | 65.0 (52.0 - 78.0) | 63.0 (52.0 - 75.0) | 61.0 (50.0 - 71.0) | 58.0 (48.0 - 68.0) | <0.001 |
| **Female, N. (%)** | 5241 (58.6) | 26807 (49.4) | 20086 (46.3) | 12113 (53.1) | 6432 (61.3) | 6106 (66.5) | <0.001 |
| **Black, N. (%)** | 2584 (28.9) | 13757 (25.4) | 10250 (23.6) | 5961 (26.1) | 3349 (31.9) | 3461 (37.7) | <0.001 |
| **White, N. (%)** | 3083 (34.4) | 18841 (34.7) | 14230 (32.8) | 6943 (30.4) | 2812 (26.8) | 2332 (25.4) | <0.001 |
| **Comorbidities** | | | | | | | |
| **CAD, N. (%)** | 1477 (16.5) | 11,385 (21.0) | 11,306 (26.0) | 6396 (28.0) | 2807 (26.7) | 2369 (25.8) | <0.001 |
| **CHF, N. (%)** | 1595 (17.8) | 10,584 (19.5) | 9397 (21.6) | 5472 (24.0) | 2890 (27.5) | 3057 (33.3) | <0.001 |
| **DM, N. (%)** | 2182 (24.4) | 16,060 (29.6) | 15,822 (36.4) | 9738 (42.7) | 5140 (49.0) | 4854 (52.9) | <0.001 |
| **HTN, N. (%)** | 3786 (42.3) | 24,429 (45.1) | 22,051 (50.8) | 12646 (55.4) | 6337 (60.4) | 5647 (61.5) | <0.001 |
| **CKD, N. (%)** | 1309 (14.6) | 9239 (17.0) | 7978 (18.4) | 4406 (19.3) | 2148 (20.5) | 2070 (22.5) | <0.001 |
| **COPD, N. (%)** | 1587 (17.7) | 6708 (12.4) | 5314 (12.2) | 3156 (13.8) | 1905 (18.1) | 1943 (21.2) | <0.001 |
| **Cancer, N. (%)** | 2987 (33.4) | 16,808 (31.0) | 12,075 (27.8) | 5981 (26.2) | 2632 (25.1) | 2108 (23.0) | <0.001 |
| **Past or present smoking, N. (%)** | 3982 (44.5) | 22,988 (42.4) | 18,370 (42.3) | 9823 (43.1) | 4615 (44.0) | 4189 (45.6) | <0.001 |
| **Mortality** | | | | | | | |
| **Mortality, N. (%)** | 427 (4.8) | 1371 (2.5) | 778 (1.8) | 386 (1.7) | 129 (1.2) | 119 (1.3) | <0.001 |

Abbreviations: BMI - body mass index; IQR - interquartile range; CAD – Coronary artery disease; CHF – Congestive heart failure; DM – Diabetes mellitus; HTN – hypertension; CKD – Chronic kidney disease; COPD – Chronic obstructive pulmonary disease.**Supplementary Table 2:** Clinical and demographic characteristics of patients in the during-COVID-19 cohort, stratified by BMI groups.

|  | **<18.5 kg/m^2^ (n = 1825, 6.0%)** | **18.5 - 25 kg/m^2^ (n = 10532, 34.9%)** | **25 - 30 kg/m^2^ (n = 9010, 29.8%)** | **30 - 35 kg/m^2^ (n = 4763, 15.8%)** | **35 - 40 kg/m^2^ (n = 2242, 7.4%)** | **≥40 kg/m^2^ (n = 1818, 6.0%)** | **P value** |
| --- | --- | --- | --- | --- | --- | --- | --- |
| **Demographics** | | | | | | | |
| **Age, median (IQR), y** | 71.0 (57.0 - 84.0) | 69.0 (55.0 - 82.0) | 66.0 (55.0 - 78.0) | 64.0 (53.0 - 74.0) | 61.5 (50.0 - 72.0) | 59.0 (46.0 - 69.0) | <0.001 |
| **Female, N. (%)** | 1001 (54.8) | 4862 (46.2) | 3870 (43.0) | 2367 (49.7) | 1290 (57.5) | 1133 (62.3) | <0.001 |
| **Black, N. (%)** | 624 (34.2) | 3064 (29.1) | 2458 (27.3) | 1437 (30.2) | 796 (35.5) | 774 (42.6) | <0.001 |
| **White, N. (%)** | 599 (32.8) | 3513 (33.4) | 2777 (30.8) | 1426 (29.9) | 677 (30.2) | 467 (25.7) | <0.001 |
| **Comorbidities** | | | | | | | |
| **CAD, N. (%)** | 433 (23.7) | 2966 (28.2) | 2688 (29.8) | 1487 (31.2) | 709 (31.6) | 482 (26.5) | <0.001 |
| **CHF, N. (%)** | 389 (21.3) | 2446 (23.2) | 2206 (24.5) | 1258 (26.4) | 642 (28.6) | 610 (33.6) | <0.001 |
| **DM, N. (%)** | 565 (31.0) | 3759 (35.7) | 3639 (40.4) | 2192 (46.0) | 1114 (49.7) | 957 (52.6) | <0.001 |
| **HTN, N. (%)** | 945 (51.8) | 5746 (54.6) | 5188 (57.6) | 2941 (61.7) | 1402 (62.5) | 1176 (64.7) | <0.001 |
| **CKD, N. (%)** | 322 (17.6) | 2018 (19.2) | 1730 (19.2) | 947 (19.9) | 471 (21.0) | 393 (21.6) | 0.015 |
| **COPD, N. (%)** | 324 (17.8) | 1387 (13.2) | 1075 (11.9) | 679 (14.3) | 353 (15.7) | 357 (19.6) | <0.001 |
| **Cancer, N. (%)** | 687 (37.6) | 3380 (32.1) | 2641 (29.3) | 1292 (27.1) | 611 (27.3) | 457 (25.1) | <0.001 |
| **Past or present smoking, N. (%)** | 775 (42.5) | 4038 (38.3) | 3195 (35.5) | 1728 (36.3) | 818 (36.5) | 665 (36.6) | <0.001 |
| **Mortality** | | | | | | | |
| **Mortality, N. (%)** | 107 (5.9) | 360 (3.4) | 306 (3.4) | 115 (2.4) | 51 (2.3) | 51 (2.8) | <0.001 |

Abbreviations: BMI - body mass index; IQR - interquartile range; CAD – Coronary artery disease; CHF – Congestive heart failure; DM – Diabetes mellitus; HTN – hypertension; CKD – Chronic kidney disease; COPD – Chronic obstructive pulmonary disease**Supplementary Table 3A:** The ten most common primary diagnoses in the severe obesity group before the COVID-19 pandemic.

| **Diagnosis** | **Number of patients with severe obesity (%)** | **Number of patients without severe obesity (%)** | **Odds Ratio** | **Mutual Information*** | **P value** |
| --- | --- | --- | --- | --- | --- |
| Cellulitis | 693 (7.5%) | 3993 (2.9%) | 2.8 | 5.9 | <0.001 |
| Shortness of breath | 657 (7.2%) | 4560 (3.3%) | 2.3 | 4.5 | <0.001 |
| Asthma | 434 (4.7%) | 2096 (1.5%) | 3.3 | 4.3 | <0.001 |
| Heart failure | 642 (7.0%) | 4836 (3.5%) | 2.1 | 4 | <0.001 |
| Chest pain | 567 (6.2%) | 6768 (4.8%) | 1.3 | 1.2 | <0.001 |
| COPD | 254 (2.8%) | 2457 (1.8%) | 1.6 | 1 | <0.001 |
| Pulmonary embolism | 111 (1.2%) | 829 (0.6%) | 2.1 | 0.7 | <0.001 |
| Respiratory failure | 78 (0.8%) | 502 (0.4%) | 2.4 | 0.6 | <0.001 |
| Atrial fibrillation | 165 (1.8%) | 1739 (1.2%) | 1.5 | 0.5 | <0.001 |
| Back pain | 107 (1.2%) | 959 (0.7%) | 1.7 | 0.5 | <0.001 |

* Mutual information measures the statistical dependence between the severe obesity group and a given diagnosis.

**Supplementary Table 3B:** The ten most common primary diagnoses in the severe obesity group during the COVID-19 pandemic.

| **Diagnosis** | **Number of patients with severe obesity (%)** | **Number of patients without severe obesity (%)** | **Odds Ratio** | **Mutual Information*** | **P value** |
| --- | --- | --- | --- | --- | --- |
| Shortness of breath | 134 (7.4%) | 878 (3.1%) | 2.5 | 5.0 | <0.001 |
| Cellulitis | 105 (5.8%) | 618 (2.2%) | 2.8 | 4.4 | <0.001 |
| Heart failure | 130 (7.2%) | 979 (3.5%) | 2.2 | 4.1 | <0.001 |
| Asthma | 40 (2.2%) | 202 (0.7%) | 3.1 | 1.9 | <0.001 |
| COVID-19 | 155 (8.5%) | 1893 (6.7%) | 1.3 | 1.7 | 0.003 |
| COPD | 38 (2.1%) | 345 (1.2%) | 1.7 | 0.9 | 0.002 |
| Pulmonary embolism | 29 (1.6%) | 227 (0.8%) | 2 | 0.9 | <0.001 |
| Pneumonia | 131 (7.2%) | 1766 (6.2%) | 1.2 | 0.9 | 0.10 |
| Hypoxia | 40 (2.2%) | 410 (1.4%) | 1.5 | 0.7 | 0.013 |
| Respiratory failure | 32 (1.8%) | 373 (1.3%) | 1.3 | 0.4 | 0.13 |

* Mutual information measures the statistical dependence between the severe obesity group and a given diagnosis.

**Supplementary Figure** **1**: Pearson correlation matrix for covariates used in the multivariable logistic regression model for the (A) pre-COVID-19 cohort and (B) during-COVID-19 cohort.

**A.**


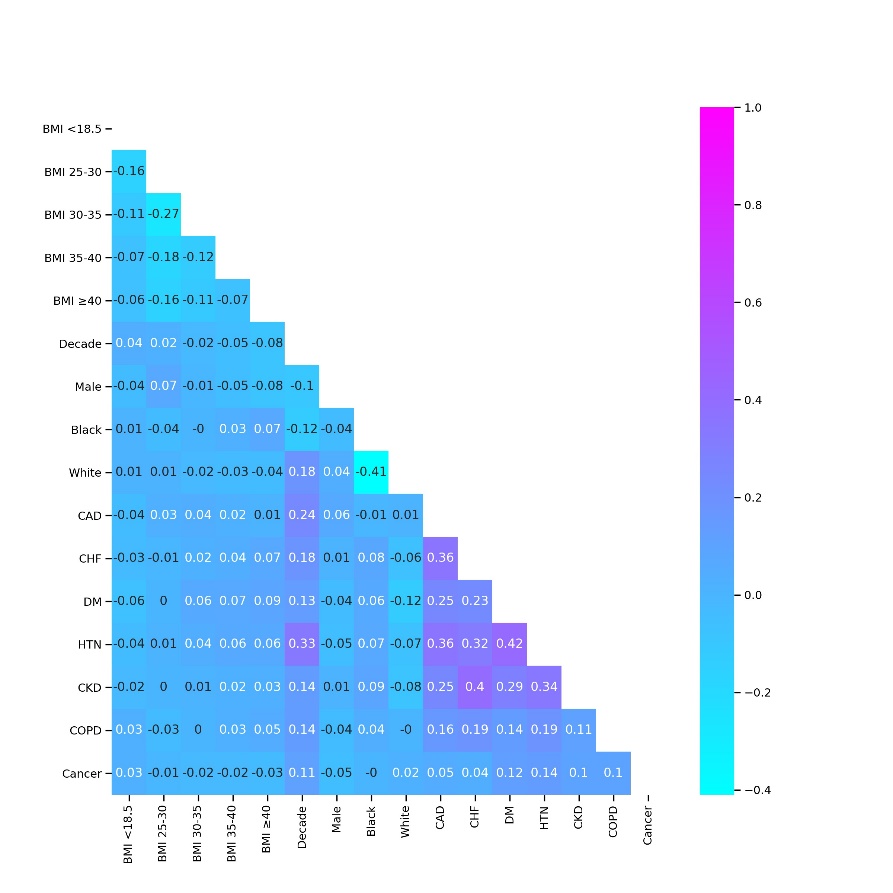


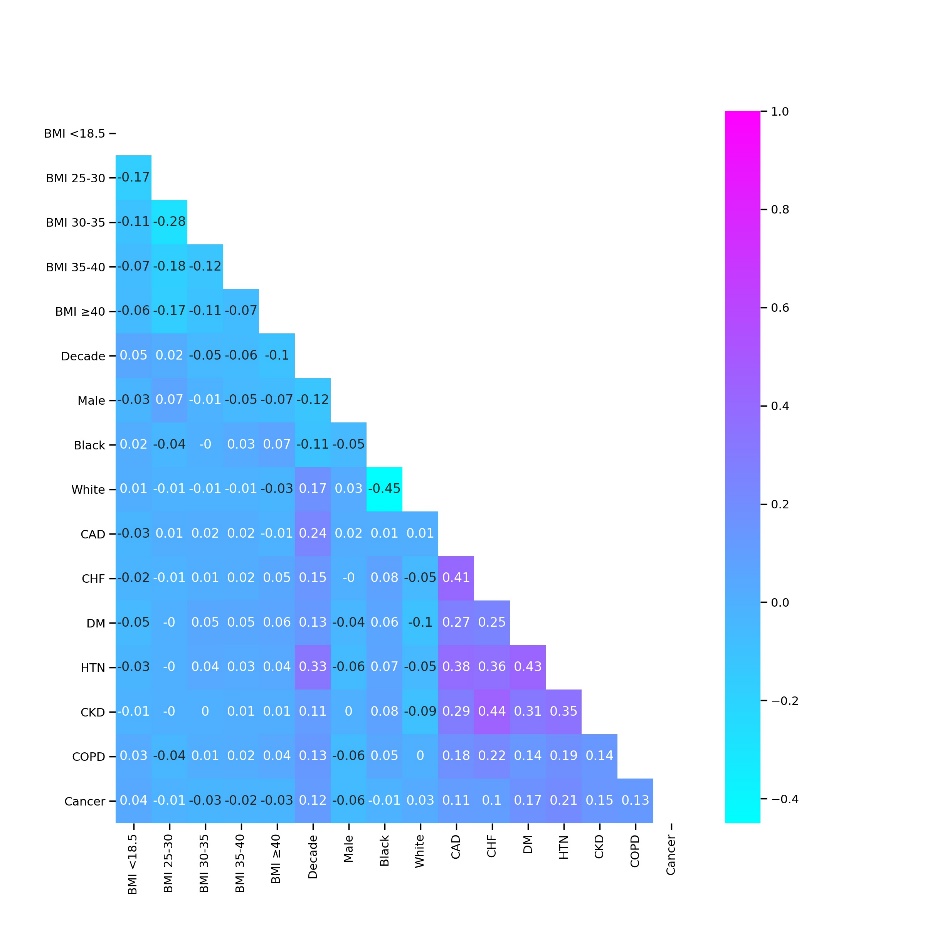
**Supplementary Figure 2:** Histogram of the BMI distribution in the study cohorts.

**B.**


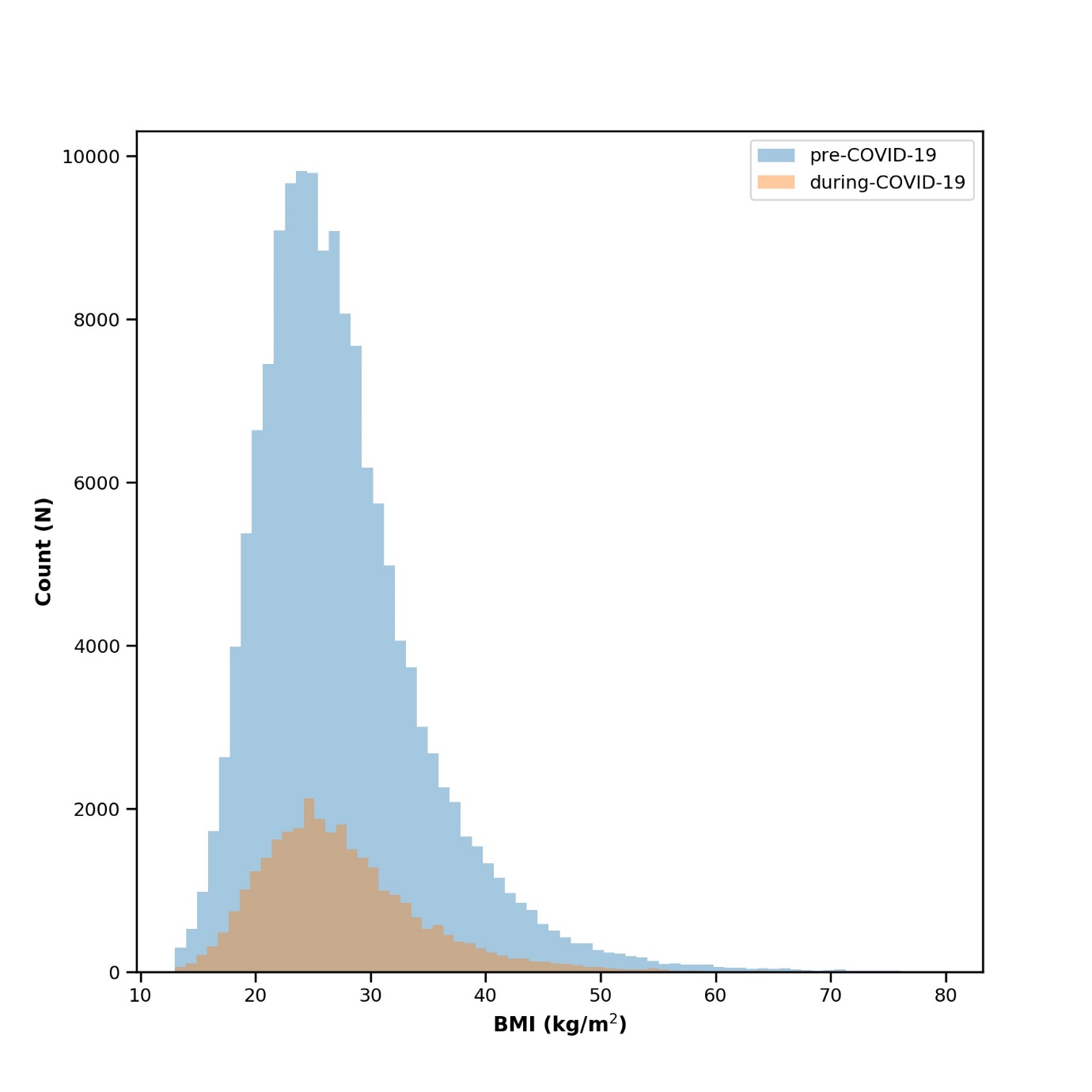


**Supplementary Figure 3**: In-hospital mortality rates according to BMI for the pre-COVID-19 cohort and the during-COVID-19 cohort for (A) patients older than fifty, (B) patients
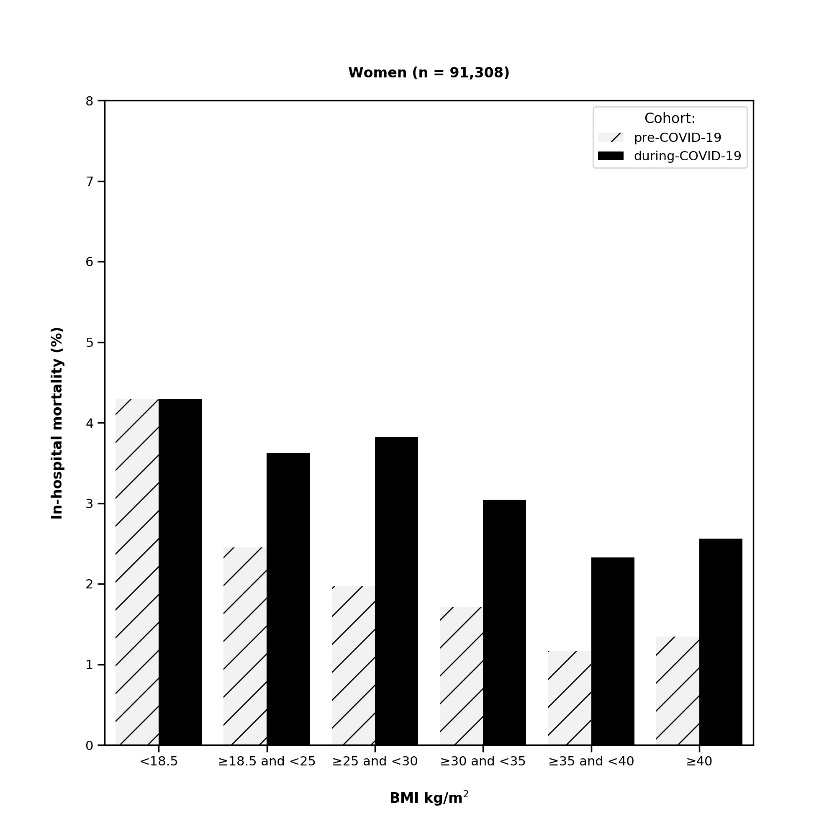
younger than fifty (C) male sex and (D)
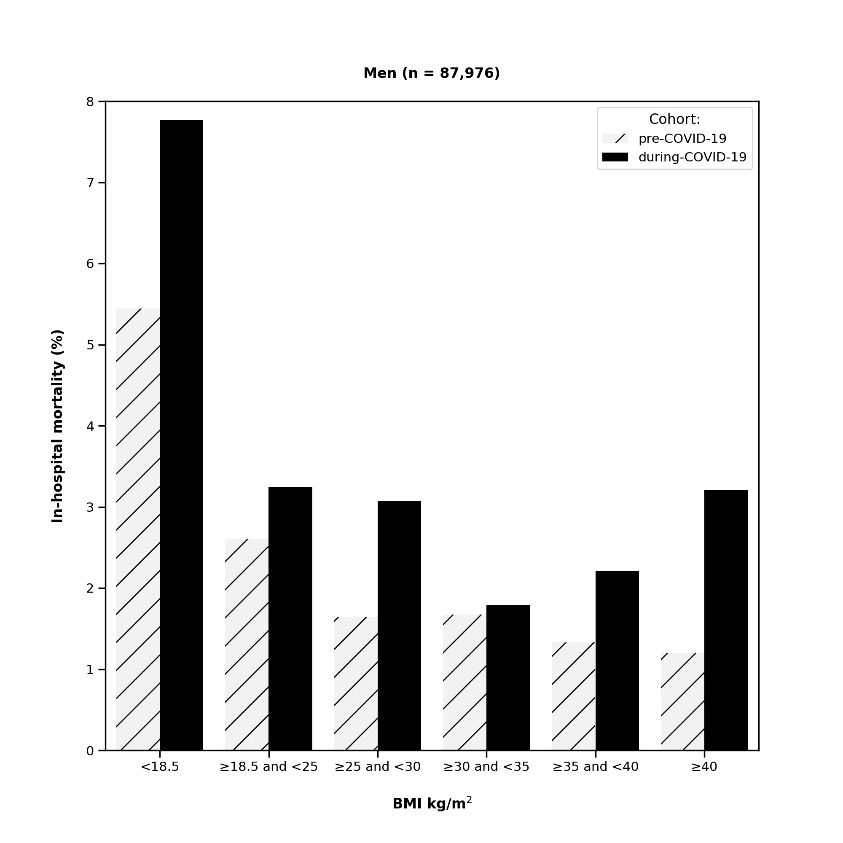

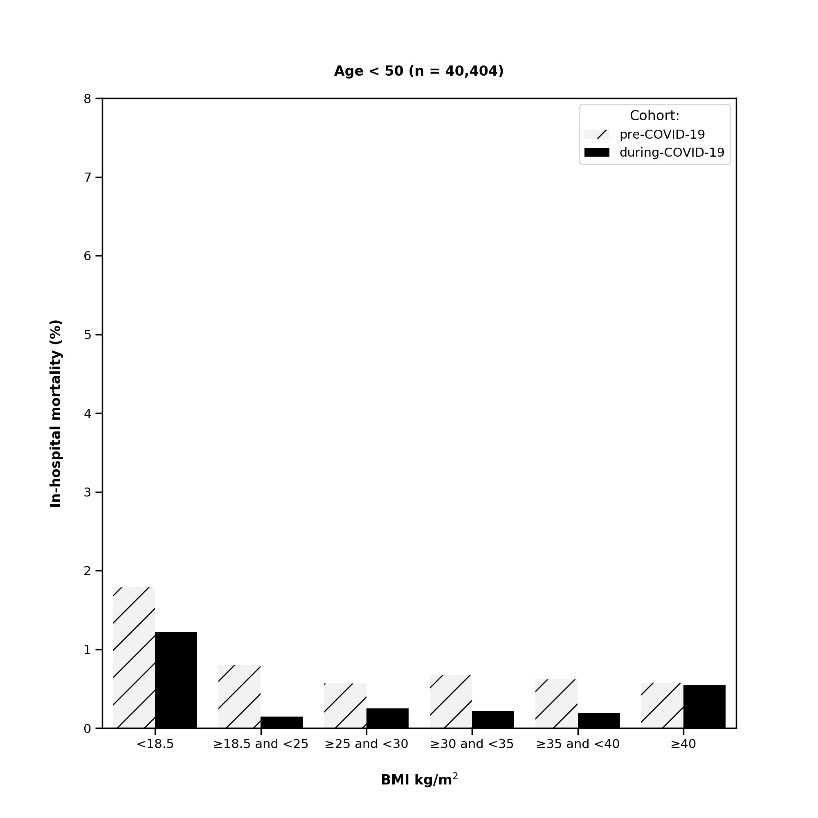

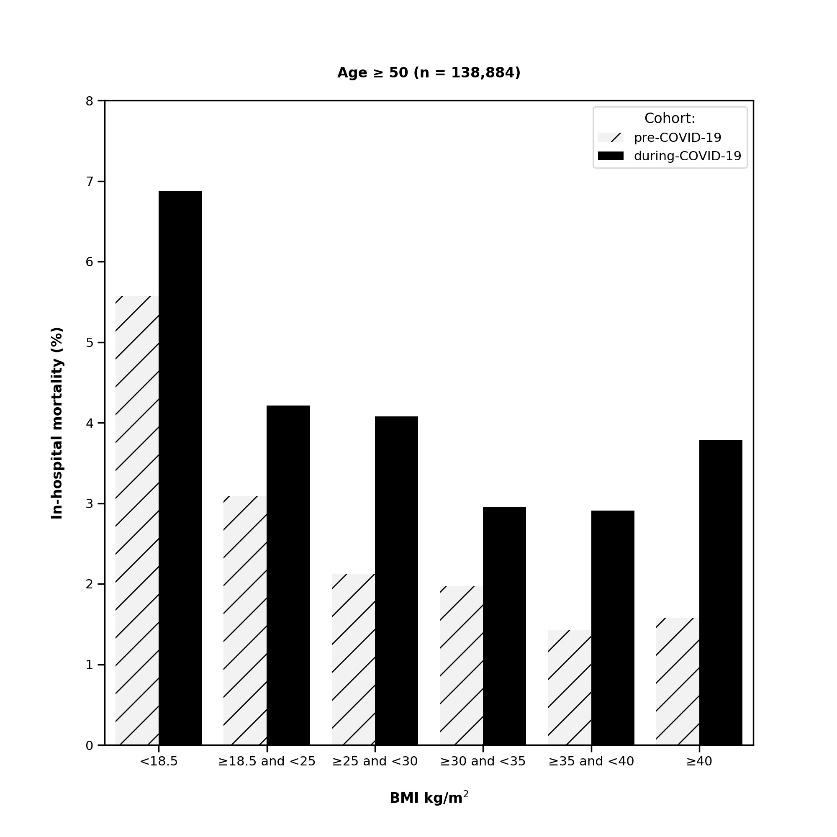
female sex.

**D.**

**C.**

**B.**

**A.**
